# A *P*,*O*,*P*′-tridentate mixed-donor scorpionate ligand: 6-[4,6-bis­(diphenyl­phosphino)-10*H*-phenoxazin-10-yl]hexan-1-ol

**DOI:** 10.1107/S1600536808030134

**Published:** 2008-09-20

**Authors:** Thashree Marimuthu, Muhammad D. Bala, Holger B. Friedrich

**Affiliations:** a School of Chemistry, University of KwaZulu-Natal, Westville Campus, Private Bag X54001, Durban 4000, South Africa

## Abstract

The title compound, C_42_H_39_NO_2_P_2_, is a *P*,*O*,*P*′-tridentate scorpionate-type ligand and has one mol­ecule in the asymmetric unit. The angles involving the P atoms range from 100.21 (7) to 104.89 (7)°. The *N*-hexa­nol group was found to be disordered and was refined over two positions with final occupancies of 0.683 (3) and 0.317 (3) which affected the C—O and C—N bond lengths. The bond lengths for C—O range from 1.402 (2) to 1.415 (2) Å and for C—N from 1.410 (2) to 1.448 (3) Å for the major disorder component; the corresponding ranges for the minor disorder component are 1.429 (3)–1.408 (3) and 1.474 (3)–1.474 (4) Å.

## Related literature

For scorpionate type ligands based on the nixantphos backbone, see: Marimuthu *et al.* (2008*a* ▶,*b* ▶). For scorpionate ligands, see: Pettinari, (2004 ▶); Trofimenko (1993 ▶); Leung, (2007 ▶); Mayer *et al.* (1994 ▶). For hydrogen bonding, see: Chen & Craven (1995 ▶); Monge *et al.* (1978 ▶). For details of the synthesis, see: Reymond *et al.* (1996 ▶); Van der Veen *et al.* (2000 ▶). For a related structure, see: Osiński *et al.* (2005 ▶).
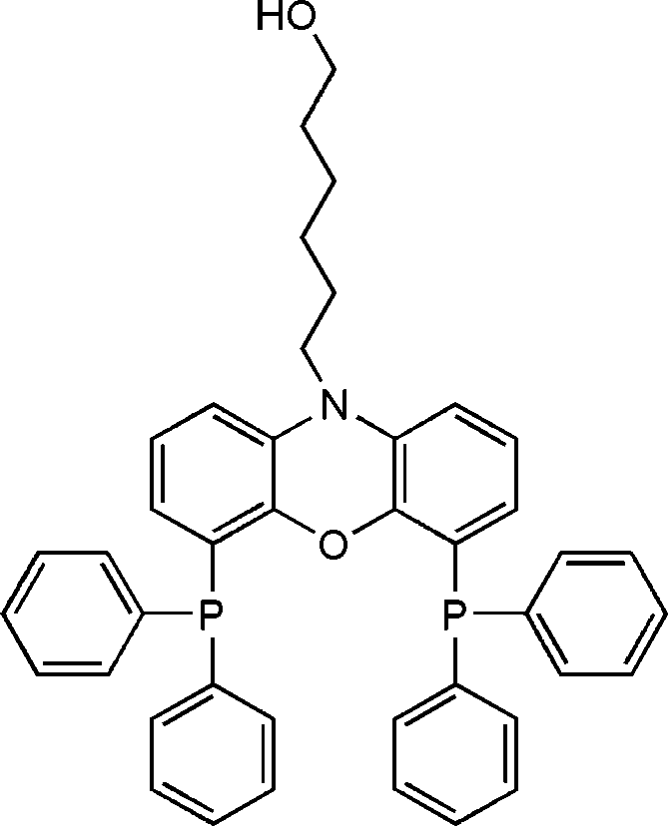


## Experimental

### 

#### Crystal data

C_42_H_39_NO_2_P_2_
*M* *_r_* = 651.68
Triclinic, 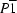

*a* = 10.4258 (2) Å
*b* = 11.1402 (3) Å
*c* = 15.3590 (4) Å
α = 75.777 (1)°
β = 88.583 (1)°
γ = 79.453 (2)°
*V* = 1699.60 (7) Å^3^
*Z* = 2
Mo *K*α radiation
μ = 0.17 mm^−1^
*T* = 173 (2) K
0.51 × 0.31 × 0.29 mm

#### Data collection

Bruker APEXII  CCD area-detector diffractometer
Absorption correction: none
30269 measured reflections
8211 independent reflections
6458 reflections with *I* > 2σ(*I*)
*R*_int_ = 0.043

#### Refinement

*R*[*F* ^2^ > 2σ(*F* ^2^)] = 0.042
*wR*(*F* ^2^) = 0.122
*S* = 1.08
8211 reflections
489 parameters
115 restraints
H-atom parameters constrained
Δρ_max_ = 0.42 e Å^−3^
Δρ_min_ = −0.30 e Å^−3^

### 

Data collection: *APEX2* (Bruker, 2005 ▶); cell refinement: *SAINT-NT* (Bruker, 2005 ▶); data reduction: *SAINT-NT*; program(s) used to solve structure: *SHELXTL* (Sheldrick, 2008 ▶); program(s) used to refine structure: *SHELXTL*; molecular graphics: *PLATON* (Spek, 2003 ▶) and *ORTEP-3* (Farrugia, 1997 ▶); software used to prepare material for publication: *SHELXTL*.

## Supplementary Material

Crystal structure: contains datablocks global, I. DOI: 10.1107/S1600536808030134/rt2023sup1.cif

Structure factors: contains datablocks I. DOI: 10.1107/S1600536808030134/rt2023Isup2.hkl

Additional supplementary materials:  crystallographic information; 3D view; checkCIF report

## Figures and Tables

**Fig. 1.**
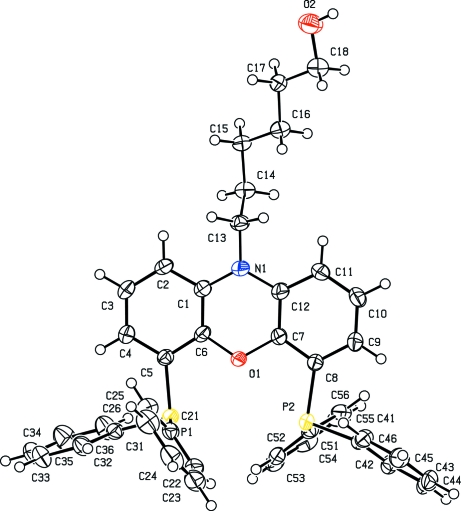
Molecular structure of the title complex. Thermal ellipsoids are shown at 50% probability levels.

## supplementary crystallographic information

### Comment

The title compound (I) was synthesized as part of our on going investigation of scorpionate type ligands based on the nixantphos backbone (Marimuthu *et al.*,2008*a*,b). Scorpionate ligands coordinate to metal centres to give unique types of coordination compounds. These compounds exhibit a characteristic type of geometry, enforced by the pincers and the third donor which comes across the plane (formed by metal and pincers) to coordinate to the metal (Pettinari, 2004). The common feature of most scorpionate ligands is that they uniformly contain a single type of donor atom, typically N– (Trofimenko, 1993), O– (Leung, 2007), or P– (Mayer *et al.*, 1994). In contrast, this research is focused on the preparation of chelating P-donor ligands based on a nixantphos backbone functionalized by various hard and soft donors that serve as the third binding site of the ligands. The funtionalized N-hexanol group was found to be disordered and was refined over two positions with final occupancies of 0.68 and 0.32. If the nixantphos moiety is considered as the 'head' of the compound (I) and the hexanol chain as its 'tail', then the packing in (I) can be described as stacked in a 'head' to 'tail' arrangement. Due to this arrangement several intermolecular interactions especially of type O2—H···O1 are observed between the 'heads' and 'tails' of adjacent molecules. The H···O1 interatomic lengths range from 2.733 to 3.346 Å. Although these are unusually long for classical hydrogen bonding (Chen and Craven, 1995; Monge *et al.*, 1978), the interactions are significant in maintaining the integrity of the disordered crystal structure. The bond angles involving the P atoms range from 100.21 (7) ° to 104.89 (7) °.

### Experimental

A two part synthesis involving: a) alkylation (Reymond *et al.*, 1996), and b) deprotection (van der Veen *et al.*, 2000) was adapted from literature. Nixantphos (0.20 g, 0.36 mmol) was dissolved in 4 ml of dry DMF to which NaH (0.22 g, 0.54 millimol, 60% dispersed in mineral oil) was added, followed by the addition of (6-bromohexyloxy)(tertbutyl)dimethylsilane (0.18 g, 0.63 millimol). The resulting mixture was stirred overnight at 100 °C. The reaction was cooled to room temperature and hydrolysed with 10 ml of water. The organic phase was extracted with 4 *x* 15 ml ethyl acetate and the combined fractions dried over sodium sulfate. Thereafter the solvent was removed *in vacuo* and the residue chromatographed with 10% hexane/ethyl acetate to give the protected precursor of the title compound (I). The precursor was dissolved in 25 ml THF, and 2.5 equivalents of tetra-*n*-butylammoniumflouride trihydrate was added. The reaction was left to stir overnight at room temperature and was followed by aqueous work-up. The resulting crude product of compound (I) was chromatographed with 20% hexane/ethyl acetate and recrystalized from a solution of dichloromethane/ethanol (1:1) to yield 23% of pale yellow crystals of pure (I). m.p. 440–441 K.

Spectroscopic analysis: ^1^H NMR (400 MHz, CDCl_3_, δ, p.p.m): = 1.67–1.24 (m, CH_2_), 3.46 (t, CH_2_OH), 3.65 (t, NCH_2_),5.97 (d, 2H; J(H,H) = 7.8 Hz), 6.41 (d, 2H; J(H,H) = 7.8 Hz), 6.64 (t, 2H; J(H,H) = 7.8 Hz), 7.20–7.18 (m, 20H).

^13^C NMR (400 MHz, CDCl_3_, δ, p.p.m): = 24.9 (CH_2_),25.8 (CH_2_), 27.0 (CH_2_), 32.9 (CH_2_), 44.8 (CH_2_), 63.0 (OCH_2_), 111.7 (CH), 123.6, 125.1 (CH), 128.1 (CH), 128.1 (CH), 128.1 (CH), 128.2 (CH), 134.0 (m, CH), 137.0 (t, C), 147.1 (t, CO).

^31^P NMR (600 MHz, CDCl_3_, δ, p.p.m): = -19.2.

MS m/*z* -[fragment]-(%): 651.2485 –[*M*] – (34%), calculated = 651.25 for C_42_H_39_NO_2_P_2_.

FTIR: cm^-1^ = 3582(s, OH), 3043(*m*), 3047(*m*), 2919(*m*), 2848(*m*), 1943(*m*), 1874(*m*), 1803(*m*), 1574(*s*), 1545(*s*), 1550(w), 1459, 1414(*s*), 1375(*s*), 1269(w), 1224(*m*), 1176(*s*), 1089 (CO), 742(*s*), 692(*s*).

### Refinement

Non-hydrogen atoms were first refined isotropically followed by anisotropic refinement by full matrix least-squares calculations based on F2 using *SHELXTL*. Hydrogen atoms were first located in the difference map then positioned geometrically and allowed to ride on their respective parent atoms. The N-hexanol group was found to be disordered and was refined over two positions using a combination of SADI, SAME, DELU and SIMU restraints, with final occupancies of 0.683 (3) and 0.317 (3).

All hydrogen atoms were first located in the difference map then positioned geometrically and allowed to ride on their respective parent atoms (C — H = 0.95 - 0.99 Å) with *U*_iso_(H) = 1.2 *U*_eq_(C) for aryl H or 1.5 *U*_eq_(C) for alkyl.

### Crystal data

**Table:**

C_42_H_39_NO_2_P_2_	*Z* = 2
*M**_r_* = 651.68	*F*(000) = 688
Triclinic, *P*1	*D*_x_ = 1.273 Mg m^−^^3^
Hall symbol: -P 1	Mo *K*α radiation, λ = 0.71073 Å
*a* = 10.4258 (2) Å	Cell parameters from 5800 reflections
*b* = 11.1402 (3) Å	θ = 2.4–28.4°
*c* = 15.3590 (4) Å	µ = 0.17 mm^−^^1^
α = 75.777 (1)°	*T* = 173 K
β = 88.583 (1)°	Prismic, yellow
γ = 79.453 (2)°	0.51 × 0.31 × 0.29 mm
*V* = 1699.60 (7) Å^3^	

### Data collection

**Table:**

Bruker SMART CCD area-detector diffractometer	6458 reflections with *I* > 2σ(*I*)
Radiation source: fine-focus sealed tube	*R*_int_ = 0.043
graphite	θ_max_ = 28.0°, θ_min_ = 1.4°
φ and ω scans	*h* = −13→13
30269 measured reflections	*k* = −14→14
8211 independent reflections	*l* = −20→19

### Refinement

**Table:**

Refinement on *F*^2^	Primary atom site location: structure-invariant direct methods
Least-squares matrix: full	Secondary atom site location: difference Fourier map
*R*[*F*^2^ > 2σ(*F*^2^)] = 0.042	Hydrogen site location: inferred from neighbouring sites
*wR*(*F*^2^) = 0.122	H-atom parameters constrained
*S* = 1.08	*w* = 1/[σ^2^(*F*_o_^2^) + (0.0654*P*)^2^ + 0.218*P*] where *P* = (*F*_o_^2^ + 2*F*_c_^2^)/3
8211 reflections	(Δ/σ)_max_ = 0.001
489 parameters	Δρ_max_ = 0.42 e Å^−^^3^
115 restraints	Δρ_min_ = −0.30 e Å^−^^3^

### Special details

**Table:**

Geometry. All e.s.d.'s (except the e.s.d. in the dihedral angle between two l.s. planes) are estimated using the full covariance matrix. The cell e.s.d.'s are taken into account individually in the estimation of e.s.d.'s in distances, angles and torsion angles; correlations between e.s.d.'s in cell parameters are only used when they are defined by crystal symmetry. An approximate (isotropic) treatment of cell e.s.d.'s is used for estimating e.s.d.'s involving l.s. planes.
Refinement. Refinement of *F*^2^ against ALL reflections. The weighted *R*-factor *wR* and goodness of fit *S* are based on *F*^2^, conventional *R*-factors *R* are based on *F*, with *F* set to zero for negative *F*^2^. The threshold expression of *F*^2^ > σ(*F*^2^) is used only for calculating *R*-factors(gt) *etc*. and is not relevant to the choice of reflections for refinement. *R*-factors based on *F*^2^ are statistically about twice as large as those based on *F*, and *R*- factors based on ALL data will be even larger.

### Fractional atomic coordinates and isotropic or equivalent isotropic displacement parameters (Å^2^)

**Table:**

	*x*	*y*	*z*	*U*_iso_*/*U*_eq_	Occ. (<1)
C1	0.10850 (18)	0.95659 (15)	0.14870 (12)	0.0403 (4)	
C2	0.04581 (16)	1.07476 (14)	0.15453 (11)	0.0341 (4)	
H2	0.0560	1.1469	0.1087	0.041*	
C3	−0.03150 (17)	1.08802 (15)	0.22679 (12)	0.0386 (4)	
H3	−0.0729	1.1698	0.2307	0.046*	
C4	−0.04999 (17)	0.98523 (15)	0.29330 (12)	0.0363 (4)	
H4	−0.1037	0.9965	0.3426	0.044*	
C5	0.00996 (14)	0.86440 (13)	0.28850 (10)	0.0263 (3)	
C6	0.08763 (16)	0.85335 (14)	0.21642 (12)	0.0348 (4)	
C7	0.21739 (16)	0.71321 (15)	0.14128 (11)	0.0338 (4)	
C8	0.26737 (14)	0.58992 (14)	0.14232 (10)	0.0261 (3)	
C9	0.34299 (16)	0.56684 (16)	0.07008 (11)	0.0350 (4)	
H9	0.3794	0.4827	0.0687	0.042*	
C10	0.36543 (17)	0.66473 (17)	0.00077 (11)	0.0381 (4)	
H10	0.4169	0.6474	−0.0481	0.046*	
C11	0.31406 (16)	0.78831 (16)	0.00116 (11)	0.0347 (4)	
H11	0.3297	0.8551	−0.0474	0.042*	
C12	0.23980 (18)	0.81449 (15)	0.07248 (12)	0.0393 (4)	
C13	0.2513 (3)	1.0437 (2)	0.02672 (16)	0.0311 (6)	0.683 (3)
H13A	0.3408	1.0148	0.0080	0.037*	0.683 (3)
H13B	0.2561	1.1077	0.0607	0.037*	0.683 (3)
C14	0.1672 (2)	1.1065 (2)	−0.05704 (17)	0.0350 (6)	0.683 (3)
H14A	0.0766	1.1336	−0.0394	0.042*	0.683 (3)
H14B	0.1658	1.0449	−0.0936	0.042*	0.683 (3)
C15	0.2183 (2)	1.2209 (2)	−0.11373 (16)	0.0357 (6)	0.683 (3)
H15A	0.1520	1.2685	−0.1606	0.043*	0.683 (3)
H15B	0.2304	1.2770	−0.0748	0.043*	0.683 (3)
C16	0.3457 (4)	1.1862 (4)	−0.1583 (3)	0.0408 (10)	0.683 (3)
H16A	0.4121	1.1400	−0.1111	0.049*	0.683 (3)
H16B	0.3338	1.1283	−0.1959	0.049*	0.683 (3)
C17	0.3969 (4)	1.2969 (4)	−0.2162 (3)	0.0381 (11)	0.683 (3)
H17A	0.4147	1.3514	−0.1775	0.046*	0.683 (3)
H17B	0.3276	1.3467	−0.2602	0.046*	0.683 (3)
C13B	0.1715 (5)	1.0427 (5)	−0.0122 (3)	0.0339 (12)	0.317 (3)
H13C	0.0917	1.1085	−0.0222	0.041*	0.317 (3)
H13D	0.1896	1.0151	−0.0685	0.041*	0.317 (3)
C14B	0.2856 (6)	1.0974 (6)	0.0113 (4)	0.0476 (15)	0.317 (3)
H14C	0.3653	1.0317	0.0193	0.057*	0.317 (3)
H14D	0.2686	1.1205	0.0693	0.057*	0.317 (3)
C15B	0.3105 (7)	1.2130 (5)	−0.0593 (4)	0.0532 (17)	0.317 (3)
H15C	0.2255	1.2677	−0.0802	0.064*	0.317 (3)
H15D	0.3604	1.2610	−0.0306	0.064*	0.317 (3)
C16B	0.3839 (9)	1.1846 (9)	−0.1399 (5)	0.045 (2)	0.317 (3)
H16C	0.3285	1.1491	−0.1747	0.054*	0.317 (3)
H16D	0.4633	1.1206	−0.1193	0.054*	0.317 (3)
C17B	0.4226 (10)	1.3013 (9)	−0.1998 (7)	0.045 (3)	0.317 (3)
H17C	0.3443	1.3578	−0.2316	0.054*	0.317 (3)
H17D	0.4618	1.3473	−0.1630	0.054*	0.317 (3)
C18	0.51909 (17)	1.26407 (17)	−0.26682 (12)	0.0400 (4)	
H18A	0.5914	1.2174	−0.2242	0.048*	
H18B	0.5038	1.2105	−0.3069	0.048*	
C21	0.14465 (15)	0.69008 (15)	0.43719 (10)	0.0297 (3)	
C22	0.20133 (17)	0.56556 (16)	0.47588 (11)	0.0374 (4)	
H22	0.1618	0.4991	0.4667	0.045*	
C23	0.31469 (19)	0.5377 (2)	0.52756 (12)	0.0483 (5)	
H23	0.3525	0.4524	0.5536	0.058*	
C24	0.37245 (18)	0.6324 (2)	0.54126 (13)	0.0506 (5)	
H24	0.4510	0.6127	0.5761	0.061*	
C25	0.31694 (18)	0.7565 (2)	0.50468 (14)	0.0477 (5)	
H25	0.3559	0.8221	0.5158	0.057*	
C26	0.20443 (16)	0.78555 (17)	0.45171 (12)	0.0390 (4)	
H26	0.1680	0.8711	0.4252	0.047*	
C31	−0.12692 (15)	0.78316 (14)	0.44558 (11)	0.0289 (3)	
C32	−0.09950 (16)	0.80924 (17)	0.52627 (11)	0.0360 (4)	
H32	−0.0115	0.7957	0.5467	0.043*	
C33	−0.19952 (19)	0.85480 (18)	0.57712 (13)	0.0447 (4)	
H33	−0.1796	0.8720	0.6323	0.054*	
C34	−0.32668 (19)	0.87525 (19)	0.54871 (13)	0.0488 (5)	
H34	−0.3948	0.9059	0.5842	0.059*	
C35	−0.35526 (18)	0.8513 (2)	0.46878 (15)	0.0544 (5)	
H35	−0.4433	0.8669	0.4481	0.065*	
C36	−0.25611 (17)	0.80432 (19)	0.41813 (13)	0.0443 (4)	
H36	−0.2771	0.7862	0.3634	0.053*	
C41	0.35700 (14)	0.33337 (13)	0.22967 (10)	0.0259 (3)	
C42	0.32873 (17)	0.22495 (15)	0.21177 (11)	0.0332 (4)	
H42	0.2419	0.2223	0.1960	0.040*	
C43	0.42648 (18)	0.11970 (16)	0.21672 (12)	0.0401 (4)	
H43	0.4058	0.0459	0.2043	0.048*	
C44	0.55208 (18)	0.12183 (16)	0.23939 (12)	0.0402 (4)	
H44	0.6182	0.0496	0.2431	0.048*	
C45	0.58230 (17)	0.22873 (16)	0.25679 (12)	0.0384 (4)	
H45	0.6695	0.2305	0.2721	0.046*	
C46	0.48592 (15)	0.33387 (15)	0.25208 (11)	0.0327 (4)	
H46	0.5078	0.4073	0.2643	0.039*	
C51	0.08077 (14)	0.43856 (13)	0.20881 (11)	0.0278 (3)	
C52	−0.01832 (16)	0.43793 (14)	0.27117 (12)	0.0335 (4)	
H52	−0.0006	0.4460	0.3296	0.040*	
C53	−0.14243 (16)	0.42574 (15)	0.24918 (13)	0.0397 (4)	
H53	−0.2093	0.4257	0.2924	0.048*	
C54	−0.16890 (17)	0.41373 (16)	0.16488 (13)	0.0416 (4)	
H54	−0.2546	0.4074	0.1493	0.050*	
C55	−0.07114 (17)	0.41088 (16)	0.10282 (12)	0.0381 (4)	
H55	−0.0891	0.4002	0.0451	0.046*	
C56	0.05286 (16)	0.42354 (15)	0.12440 (11)	0.0334 (4)	
H56	0.1196	0.4220	0.0812	0.040*	
N1	0.2074 (2)	0.93759 (18)	0.08664 (16)	0.0336 (6)	0.683 (3)
N1B	0.1493 (5)	0.9347 (3)	0.0605 (2)	0.0303 (12)	0.317 (3)
O1	0.1616 (2)	0.73286 (17)	0.22274 (13)	0.0313 (5)	0.683 (3)
O1B	0.1088 (4)	0.7331 (3)	0.1957 (3)	0.0310 (11)	0.317 (3)
O2	0.55085 (14)	1.37799 (13)	−0.31765 (9)	0.0533 (4)	
H2A	0.6155	1.3624	−0.3495	0.080*	
P1	−0.00697 (4)	0.71914 (4)	0.37224 (3)	0.02700 (11)	
P2	0.23648 (4)	0.46903 (3)	0.24223 (3)	0.02641 (11)	

### Atomic displacement parameters (Å^2^)

**Table:**

	*U*^11^	*U*^22^	*U*^33^	*U*^12^	*U*^13^	*U*^23^
C1	0.0484 (10)	0.0244 (8)	0.0439 (10)	−0.0034 (7)	0.0186 (8)	−0.0049 (7)
C2	0.0402 (9)	0.0205 (7)	0.0384 (9)	−0.0043 (6)	0.0050 (7)	−0.0023 (6)
C3	0.0459 (10)	0.0220 (7)	0.0445 (10)	0.0017 (7)	0.0055 (8)	−0.0081 (7)
C4	0.0406 (9)	0.0274 (8)	0.0384 (9)	0.0002 (7)	0.0085 (7)	−0.0088 (7)
C5	0.0253 (7)	0.0235 (7)	0.0288 (8)	−0.0030 (6)	0.0007 (6)	−0.0053 (6)
C6	0.0393 (9)	0.0198 (7)	0.0409 (9)	0.0003 (6)	0.0120 (7)	−0.0045 (7)
C7	0.0366 (9)	0.0267 (8)	0.0364 (9)	−0.0040 (6)	0.0157 (7)	−0.0074 (7)
C8	0.0260 (7)	0.0249 (7)	0.0275 (8)	−0.0038 (6)	0.0012 (6)	−0.0075 (6)
C9	0.0390 (9)	0.0311 (8)	0.0328 (9)	0.0021 (7)	0.0051 (7)	−0.0106 (7)
C10	0.0397 (9)	0.0426 (9)	0.0305 (9)	−0.0024 (7)	0.0097 (7)	−0.0109 (7)
C11	0.0372 (9)	0.0347 (8)	0.0301 (8)	−0.0071 (7)	0.0086 (7)	−0.0044 (7)
C12	0.0458 (10)	0.0259 (8)	0.0433 (10)	−0.0056 (7)	0.0181 (8)	−0.0055 (7)
C13	0.0376 (14)	0.0273 (12)	0.0290 (12)	−0.0123 (10)	0.0002 (10)	−0.0036 (9)
C14	0.0378 (13)	0.0321 (13)	0.0327 (13)	−0.0079 (10)	−0.0005 (10)	−0.0024 (10)
C15	0.0464 (14)	0.0261 (11)	0.0297 (12)	−0.0035 (10)	0.0017 (10)	0.0000 (9)
C16	0.052 (2)	0.0308 (15)	0.0396 (17)	−0.0127 (14)	0.0084 (15)	−0.0063 (13)
C17	0.058 (2)	0.0271 (15)	0.0310 (17)	−0.0115 (14)	0.0083 (16)	−0.0093 (12)
C13B	0.045 (3)	0.029 (3)	0.027 (3)	−0.009 (2)	0.006 (2)	−0.004 (2)
C14B	0.055 (3)	0.049 (3)	0.049 (3)	−0.028 (3)	0.013 (3)	−0.018 (2)
C15B	0.069 (4)	0.045 (3)	0.057 (3)	−0.031 (3)	0.026 (3)	−0.023 (2)
C16B	0.057 (5)	0.037 (3)	0.050 (4)	−0.022 (3)	0.014 (3)	−0.019 (3)
C17B	0.064 (5)	0.041 (4)	0.039 (4)	−0.025 (3)	0.012 (4)	−0.017 (3)
C18	0.0402 (9)	0.0393 (9)	0.0395 (10)	−0.0092 (7)	−0.0001 (7)	−0.0059 (8)
C21	0.0262 (7)	0.0312 (8)	0.0290 (8)	−0.0011 (6)	0.0053 (6)	−0.0057 (6)
C22	0.0415 (9)	0.0339 (9)	0.0318 (9)	0.0008 (7)	0.0040 (7)	−0.0044 (7)
C23	0.0465 (11)	0.0516 (11)	0.0336 (10)	0.0133 (9)	−0.0009 (8)	−0.0020 (8)
C24	0.0333 (9)	0.0767 (15)	0.0350 (10)	0.0043 (9)	−0.0031 (8)	−0.0111 (10)
C25	0.0340 (9)	0.0641 (13)	0.0479 (11)	−0.0133 (9)	0.0014 (8)	−0.0162 (10)
C26	0.0308 (8)	0.0389 (9)	0.0453 (10)	−0.0067 (7)	0.0012 (7)	−0.0066 (8)
C31	0.0263 (7)	0.0265 (7)	0.0324 (8)	−0.0051 (6)	0.0054 (6)	−0.0049 (6)
C32	0.0313 (8)	0.0427 (9)	0.0333 (9)	−0.0058 (7)	0.0034 (7)	−0.0087 (7)
C33	0.0479 (11)	0.0519 (11)	0.0355 (10)	−0.0082 (9)	0.0119 (8)	−0.0147 (8)
C34	0.0414 (10)	0.0536 (11)	0.0462 (11)	−0.0011 (8)	0.0195 (9)	−0.0098 (9)
C35	0.0274 (9)	0.0783 (15)	0.0539 (12)	−0.0019 (9)	0.0074 (8)	−0.0157 (11)
C36	0.0285 (9)	0.0632 (12)	0.0427 (10)	−0.0077 (8)	0.0024 (7)	−0.0167 (9)
C41	0.0309 (8)	0.0223 (7)	0.0228 (7)	−0.0006 (6)	0.0019 (6)	−0.0055 (6)
C42	0.0385 (9)	0.0286 (8)	0.0331 (9)	−0.0023 (6)	−0.0054 (7)	−0.0110 (7)
C43	0.0545 (11)	0.0266 (8)	0.0395 (10)	0.0015 (7)	−0.0046 (8)	−0.0146 (7)
C44	0.0431 (10)	0.0333 (9)	0.0378 (9)	0.0110 (7)	0.0022 (8)	−0.0099 (7)
C45	0.0295 (8)	0.0385 (9)	0.0420 (10)	0.0003 (7)	0.0021 (7)	−0.0050 (8)
C46	0.0323 (8)	0.0280 (8)	0.0368 (9)	−0.0044 (6)	0.0014 (7)	−0.0067 (7)
C51	0.0272 (7)	0.0208 (7)	0.0320 (8)	−0.0009 (6)	0.0008 (6)	−0.0027 (6)
C52	0.0372 (9)	0.0241 (7)	0.0385 (9)	−0.0036 (6)	0.0081 (7)	−0.0087 (7)
C53	0.0324 (9)	0.0288 (8)	0.0566 (12)	−0.0050 (7)	0.0129 (8)	−0.0094 (8)
C54	0.0313 (9)	0.0296 (8)	0.0584 (12)	−0.0061 (7)	−0.0049 (8)	0.0002 (8)
C55	0.0410 (10)	0.0333 (9)	0.0353 (9)	−0.0093 (7)	−0.0087 (7)	0.0032 (7)
C56	0.0347 (8)	0.0316 (8)	0.0296 (8)	−0.0061 (7)	0.0011 (7)	0.0003 (7)
N1	0.0397 (13)	0.0247 (10)	0.0343 (12)	−0.0076 (9)	0.0098 (11)	−0.0031 (8)
N1B	0.034 (3)	0.022 (2)	0.033 (3)	−0.0073 (18)	0.013 (2)	−0.0041 (18)
O1	0.0354 (12)	0.0214 (8)	0.0294 (11)	0.0051 (8)	0.0100 (9)	−0.0005 (7)
O1B	0.027 (2)	0.0205 (18)	0.038 (3)	0.0042 (17)	0.0124 (19)	0.0000 (17)
O2	0.0518 (8)	0.0506 (8)	0.0500 (8)	−0.0079 (6)	0.0098 (6)	−0.0001 (6)
P1	0.0266 (2)	0.02380 (19)	0.0298 (2)	−0.00413 (15)	0.00407 (15)	−0.00582 (16)
P2	0.0285 (2)	0.02239 (19)	0.0277 (2)	−0.00139 (14)	0.00210 (15)	−0.00764 (15)

### Geometric parameters (Å, °)

**Table:**

C1—C2	1.381 (2)	C16B—H16D	0.9900
C1—C6	1.394 (2)	C17B—C18	1.500 (5)
C1—N1	1.410 (2)	C17B—H17C	0.9900
C1—N1B	1.474 (3)	C17B—H17D	0.9900
C2—C3	1.377 (2)	C18—O2	1.408 (2)
C2—H2	0.9500	C18—H18A	0.9900
C3—C4	1.374 (2)	C18—H18B	0.9900
C3—H3	0.9500	C21—C26	1.391 (2)
C4—C5	1.395 (2)	C21—C22	1.392 (2)
C4—H4	0.9500	C21—P1	1.8250 (16)
C5—C6	1.372 (2)	C22—C23	1.384 (3)
C5—P1	1.8370 (15)	C22—H22	0.9500
C6—O1	1.402 (2)	C23—C24	1.366 (3)
C6—O1B	1.429 (3)	C23—H23	0.9500
C7—C8	1.373 (2)	C24—C25	1.380 (3)
C7—C12	1.394 (2)	C24—H24	0.9500
C7—O1B	1.408 (3)	C25—C26	1.386 (2)
C7—O1	1.415 (2)	C25—H25	0.9500
C8—C9	1.393 (2)	C26—H26	0.9500
C8—P2	1.8414 (15)	C31—C36	1.382 (2)
C9—C10	1.374 (2)	C31—C32	1.388 (2)
C9—H9	0.9500	C31—P1	1.8275 (16)
C10—C11	1.384 (2)	C32—C33	1.384 (2)
C10—H10	0.9500	C32—H32	0.9500
C11—C12	1.385 (2)	C33—C34	1.367 (3)
C11—H11	0.9500	C33—H33	0.9500
C12—N1	1.420 (2)	C34—C35	1.369 (3)
C12—N1B	1.464 (3)	C34—H34	0.9500
C13—N1	1.448 (3)	C35—C36	1.382 (3)
C13—C14	1.517 (3)	C35—H35	0.9500
C13—H13A	0.9900	C36—H36	0.9500
C13—H13B	0.9900	C41—C42	1.385 (2)
C14—C15	1.532 (3)	C41—C46	1.397 (2)
C14—H14A	0.9900	C41—P2	1.8264 (14)
C14—H14B	0.9900	C42—C43	1.393 (2)
C15—C16	1.510 (4)	C42—H42	0.9500
C15—H15A	0.9900	C43—C44	1.369 (3)
C15—H15B	0.9900	C43—H43	0.9500
C16—C17	1.508 (3)	C44—C45	1.374 (3)
C16—H16A	0.9900	C44—H44	0.9500
C16—H16B	0.9900	C45—C46	1.385 (2)
C17—C18	1.510 (3)	C45—H45	0.9500
C17—H17A	0.9900	C46—H46	0.9500
C17—H17B	0.9900	C51—C52	1.391 (2)
C13B—N1B	1.474 (4)	C51—C56	1.392 (2)
C13B—C14B	1.520 (5)	C51—P2	1.8284 (16)
C13B—H13C	0.9900	C52—C53	1.384 (2)
C13B—H13D	0.9900	C52—H52	0.9500
C14B—C15B	1.524 (5)	C53—C54	1.374 (3)
C14B—H14C	0.9900	C53—H53	0.9500
C14B—H14D	0.9900	C54—C55	1.380 (3)
C15B—C16B	1.508 (5)	C54—H54	0.9500
C15B—H15C	0.9900	C55—C56	1.383 (2)
C15B—H15D	0.9900	C55—H55	0.9500
C16B—C17B	1.512 (5)	C56—H56	0.9500
C16B—H16C	0.9900	O2—H2A	0.8400
			
C2—C1—C6	117.85 (15)	C16B—C17B—H17C	109.8
C2—C1—N1	122.95 (16)	C18—C17B—H17D	109.8
C6—C1—N1	118.37 (15)	C16B—C17B—H17D	109.8
C2—C1—N1B	119.71 (19)	H17C—C17B—H17D	108.2
C6—C1—N1B	117.95 (19)	O2—C18—C17B	105.1 (4)
C3—C2—C1	120.00 (15)	O2—C18—C17	107.4 (2)
C3—C2—H2	120.0	O2—C18—H18A	110.2
C1—C2—H2	120.0	C17B—C18—H18A	98.1
C4—C3—C2	121.30 (15)	C17—C18—H18A	110.2
C4—C3—H3	119.4	O2—C18—H18B	110.2
C2—C3—H3	119.4	C17B—C18—H18B	123.7
C3—C4—C5	120.09 (15)	C17—C18—H18B	110.2
C3—C4—H4	120.0	H18A—C18—H18B	108.5
C5—C4—H4	120.0	C26—C21—C22	118.56 (15)
C6—C5—C4	117.69 (14)	C26—C21—P1	123.49 (12)
C6—C5—P1	117.70 (11)	C22—C21—P1	117.93 (13)
C4—C5—P1	124.60 (12)	C23—C22—C21	120.57 (18)
C5—C6—C1	123.05 (14)	C23—C22—H22	119.7
C5—C6—O1	114.86 (15)	C21—C22—H22	119.7
C1—C6—O1	121.33 (15)	C24—C23—C22	120.29 (18)
C5—C6—O1B	115.83 (19)	C24—C23—H23	119.9
C1—C6—O1B	117.6 (2)	C22—C23—H23	119.9
C8—C7—C12	123.22 (14)	C23—C24—C25	120.13 (18)
C8—C7—O1B	115.96 (19)	C23—C24—H24	119.9
C12—C7—O1B	117.7 (2)	C25—C24—H24	119.9
C8—C7—O1	115.02 (15)	C24—C25—C26	120.11 (19)
C12—C7—O1	120.76 (15)	C24—C25—H25	119.9
C7—C8—C9	117.48 (14)	C26—C25—H25	119.9
C7—C8—P2	117.19 (11)	C25—C26—C21	120.32 (17)
C9—C8—P2	125.24 (11)	C25—C26—H26	119.8
C10—C9—C8	120.60 (15)	C21—C26—H26	119.8
C10—C9—H9	119.7	C36—C31—C32	118.01 (15)
C8—C9—H9	119.7	C36—C31—P1	116.18 (13)
C9—C10—C11	120.92 (15)	C32—C31—P1	125.79 (12)
C9—C10—H10	119.5	C33—C32—C31	120.40 (16)
C11—C10—H10	119.5	C33—C32—H32	119.8
C10—C11—C12	119.90 (15)	C31—C32—H32	119.8
C10—C11—H11	120.1	C34—C33—C32	120.68 (18)
C12—C11—H11	120.1	C34—C33—H33	119.7
C11—C12—C7	117.88 (14)	C32—C33—H33	119.7
C11—C12—N1	122.58 (16)	C33—C34—C35	119.63 (17)
C7—C12—N1	118.43 (16)	C33—C34—H34	120.2
C11—C12—N1B	119.68 (19)	C35—C34—H34	120.2
C7—C12—N1B	118.50 (19)	C34—C35—C36	120.06 (18)
N1—C13—C14	115.7 (2)	C34—C35—H35	120.0
N1—C13—H13A	108.4	C36—C35—H35	120.0
C14—C13—H13A	108.4	C35—C36—C31	121.21 (18)
N1—C13—H13B	108.4	C35—C36—H36	119.4
C14—C13—H13B	108.4	C31—C36—H36	119.4
H13A—C13—H13B	107.4	C42—C41—C46	118.02 (14)
C13—C14—C15	111.7 (2)	C42—C41—P2	125.36 (12)
C13—C14—H14A	109.3	C46—C41—P2	116.03 (11)
C15—C14—H14A	109.3	C41—C42—C43	120.58 (16)
C13—C14—H14B	109.3	C41—C42—H42	119.7
C15—C14—H14B	109.3	C43—C42—H42	119.7
H14A—C14—H14B	107.9	C44—C43—C42	120.48 (16)
C16—C15—C14	113.2 (2)	C44—C43—H43	119.8
C16—C15—H15A	108.9	C42—C43—H43	119.8
C14—C15—H15A	108.9	C43—C44—C45	119.83 (15)
C16—C15—H15B	108.9	C43—C44—H44	120.1
C14—C15—H15B	108.9	C45—C44—H44	120.1
H15A—C15—H15B	107.7	C44—C45—C46	120.17 (16)
C17—C16—C15	114.4 (3)	C44—C45—H45	119.9
C17—C16—H16A	108.7	C46—C45—H45	119.9
C15—C16—H16A	108.7	C45—C46—C41	120.91 (15)
C17—C16—H16B	108.7	C45—C46—H46	119.5
C15—C16—H16B	108.7	C41—C46—H46	119.5
H16A—C16—H16B	107.6	C52—C51—C56	118.34 (15)
C16—C17—C18	115.4 (3)	C52—C51—P2	116.88 (12)
C16—C17—H17A	108.4	C56—C51—P2	124.67 (12)
C18—C17—H17A	108.4	C53—C52—C51	120.89 (16)
C16—C17—H17B	108.4	C53—C52—H52	119.6
C18—C17—H17B	108.4	C51—C52—H52	119.6
H17A—C17—H17B	107.5	C54—C53—C52	119.95 (16)
N1B—C13B—C14B	111.0 (5)	C54—C53—H53	120.0
N1B—C13B—H13C	109.4	C52—C53—H53	120.0
C14B—C13B—H13C	109.4	C53—C54—C55	120.06 (16)
N1B—C13B—H13D	109.4	C53—C54—H54	120.0
C14B—C13B—H13D	109.4	C55—C54—H54	120.0
H13C—C13B—H13D	108.0	C54—C55—C56	120.15 (17)
C13B—C14B—C15B	113.8 (4)	C54—C55—H55	119.9
C13B—C14B—H14C	108.8	C56—C55—H55	119.9
C15B—C14B—H14C	108.8	C55—C56—C51	120.58 (16)
C13B—C14B—H14D	108.8	C55—C56—H56	119.7
C15B—C14B—H14D	108.8	C51—C56—H56	119.7
H14C—C14B—H14D	107.7	C1—N1—C12	116.48 (16)
C16B—C15B—C14B	114.8 (5)	C1—N1—C13	120.66 (18)
C16B—C15B—H15C	108.6	C12—N1—C13	121.43 (18)
C14B—C15B—H15C	108.6	C12—N1B—C13B	119.2 (3)
C16B—C15B—H15D	108.6	C12—N1B—C1	109.9 (3)
C14B—C15B—H15D	108.6	C13B—N1B—C1	118.8 (3)
H15C—C15B—H15D	107.5	C6—O1—C7	114.79 (16)
C15B—C16B—C17B	111.7 (5)	C7—O1B—C6	113.6 (3)
C15B—C16B—H16C	109.3	C18—O2—H2A	109.5
C17B—C16B—H16C	109.3	C21—P1—C31	102.21 (7)
C15B—C16B—H16D	109.3	C21—P1—C5	100.61 (7)
C17B—C16B—H16D	109.3	C31—P1—C5	100.22 (7)
H16C—C16B—H16D	107.9	C41—P2—C51	104.89 (7)
C18—C17B—C16B	109.4 (5)	C41—P2—C8	101.27 (7)
C18—C17B—H17C	109.8	C51—P2—C8	100.21 (7)
			
C6—C1—C2—C3	1.7 (3)	C43—C44—C45—C46	0.5 (3)
N1—C1—C2—C3	−167.7 (2)	C44—C45—C46—C41	0.0 (3)
N1B—C1—C2—C3	157.4 (3)	C42—C41—C46—C45	−0.4 (2)
C1—C2—C3—C4	−1.2 (3)	P2—C41—C46—C45	171.23 (13)
C2—C3—C4—C5	0.0 (3)	C56—C51—C52—C53	−1.5 (2)
C3—C4—C5—C6	0.7 (3)	P2—C51—C52—C53	174.79 (12)
C3—C4—C5—P1	−179.70 (14)	C51—C52—C53—C54	0.2 (2)
C4—C5—C6—C1	−0.1 (3)	C52—C53—C54—C55	1.5 (2)
P1—C5—C6—C1	−179.75 (15)	C53—C54—C55—C56	−1.8 (2)
C4—C5—C6—O1	170.02 (18)	C54—C55—C56—C51	0.4 (2)
P1—C5—C6—O1	−9.6 (2)	C52—C51—C56—C55	1.3 (2)
C4—C5—C6—O1B	−158.4 (3)	P2—C51—C56—C55	−174.75 (12)
P1—C5—C6—O1B	22.0 (3)	C2—C1—N1—C12	−164.7 (2)
C2—C1—C6—C5	−1.1 (3)	C6—C1—N1—C12	26.0 (3)
N1—C1—C6—C5	168.8 (2)	N1B—C1—N1—C12	−71.4 (3)
N1B—C1—C6—C5	−157.2 (3)	C2—C1—N1—C13	1.9 (4)
C2—C1—C6—O1	−170.58 (19)	C6—C1—N1—C13	−167.5 (2)
N1—C1—C6—O1	−0.7 (3)	N1B—C1—N1—C13	95.1 (5)
N1B—C1—C6—O1	33.3 (3)	C11—C12—N1—C1	166.2 (2)
C2—C1—C6—O1B	156.8 (3)	C7—C12—N1—C1	−26.1 (3)
N1—C1—C6—O1B	−33.3 (3)	N1B—C12—N1—C1	72.3 (3)
N1B—C1—C6—O1B	0.7 (4)	C11—C12—N1—C13	−0.2 (4)
C12—C7—C8—C9	−0.6 (3)	C7—C12—N1—C13	167.4 (2)
O1B—C7—C8—C9	159.0 (3)	N1B—C12—N1—C13	−94.1 (5)
O1—C7—C8—C9	−169.19 (17)	C14—C13—N1—C1	−81.6 (3)
C12—C7—C8—P2	176.11 (14)	C14—C13—N1—C12	84.3 (3)
O1B—C7—C8—P2	−24.3 (3)	C11—C12—N1B—C13B	−22.6 (6)
O1—C7—C8—P2	7.5 (2)	C7—C12—N1B—C13B	−179.8 (4)
C7—C8—C9—C10	−0.2 (2)	N1—C12—N1B—C13B	82.0 (6)
P2—C8—C9—C10	−176.62 (13)	C11—C12—N1B—C1	−164.8 (2)
C8—C9—C10—C11	0.3 (3)	C7—C12—N1B—C1	38.0 (4)
C9—C10—C11—C12	0.5 (3)	N1—C12—N1B—C1	−60.2 (3)
C10—C11—C12—C7	−1.3 (3)	C14B—C13B—N1B—C12	−74.1 (6)
C10—C11—C12—N1	166.5 (2)	C14B—C13B—N1B—C1	64.7 (6)
C10—C11—C12—N1B	−158.6 (3)	C2—C1—N1B—C12	166.4 (2)
C8—C7—C12—C11	1.3 (3)	C6—C1—N1B—C12	−37.9 (4)
O1B—C7—C12—C11	−158.0 (3)	N1—C1—N1B—C12	61.1 (3)
O1—C7—C12—C11	169.31 (19)	C2—C1—N1B—C13B	24.1 (6)
C8—C7—C12—N1	−166.94 (19)	C6—C1—N1B—C13B	179.8 (4)
O1B—C7—C12—N1	33.8 (3)	N1—C1—N1B—C13B	−81.2 (6)
O1—C7—C12—N1	1.1 (3)	C5—C6—O1—C7	165.88 (18)
C8—C7—C12—N1B	158.9 (3)	C1—C6—O1—C7	−23.8 (3)
O1B—C7—C12—N1B	−0.3 (4)	O1B—C6—O1—C7	67.1 (4)
O1—C7—C12—N1B	−33.1 (3)	C8—C7—O1—C6	−167.55 (18)
N1—C13—C14—C15	177.5 (2)	C12—C7—O1—C6	23.5 (3)
C13—C14—C15—C16	69.7 (3)	O1B—C7—O1—C6	−68.7 (4)
C14—C15—C16—C17	178.8 (3)	C8—C7—O1B—C6	160.1 (3)
C15—C16—C17—C18	−175.8 (4)	C12—C7—O1B—C6	−39.1 (5)
N1B—C13B—C14B—C15B	−177.6 (4)	O1—C7—O1B—C6	65.0 (4)
C13B—C14B—C15B—C16B	−79.7 (8)	C5—C6—O1B—C7	−161.6 (3)
C14B—C15B—C16B—C17B	−171.5 (8)	C1—C6—O1B—C7	38.9 (5)
C15B—C16B—C17B—C18	167.7 (8)	O1—C6—O1B—C7	−66.6 (3)
C16B—C17B—C18—O2	−178.7 (7)	C26—C21—P1—C31	−70.00 (15)
C16B—C17B—C18—C17	80.3 (18)	C22—C21—P1—C31	108.41 (13)
C16—C17—C18—O2	179.2 (3)	C26—C21—P1—C5	33.01 (15)
C16—C17—C18—C17B	−97.5 (19)	C22—C21—P1—C5	−148.57 (13)
C26—C21—C22—C23	0.0 (2)	C36—C31—P1—C21	−174.17 (13)
P1—C21—C22—C23	−178.52 (13)	C32—C31—P1—C21	4.35 (16)
C21—C22—C23—C24	0.0 (3)	C36—C31—P1—C5	82.51 (14)
C22—C23—C24—C25	1.0 (3)	C32—C31—P1—C5	−98.97 (15)
C23—C24—C25—C26	−1.9 (3)	C6—C5—P1—C21	79.94 (14)
C24—C25—C26—C21	1.9 (3)	C4—C5—P1—C21	−99.67 (15)
C22—C21—C26—C25	−0.9 (3)	C6—C5—P1—C31	−175.44 (13)
P1—C21—C26—C25	177.50 (14)	C4—C5—P1—C31	4.94 (16)
C36—C31—C32—C33	0.1 (3)	C42—C41—P2—C51	−8.63 (15)
P1—C31—C32—C33	−178.37 (14)	C46—C41—P2—C51	−179.58 (12)
C31—C32—C33—C34	−0.2 (3)	C42—C41—P2—C8	−112.52 (14)
C32—C33—C34—C35	−0.5 (3)	C46—C41—P2—C8	76.54 (13)
C33—C34—C35—C36	1.3 (3)	C52—C51—P2—C41	123.64 (12)
C34—C35—C36—C31	−1.4 (3)	C56—C51—P2—C41	−60.29 (14)
C32—C31—C36—C35	0.7 (3)	C52—C51—P2—C8	−131.68 (12)
P1—C31—C36—C35	179.31 (17)	C56—C51—P2—C8	44.39 (14)
C46—C41—C42—C43	0.4 (2)	C7—C8—P2—C41	−163.73 (13)
P2—C41—C42—C43	−170.36 (13)	C9—C8—P2—C41	12.66 (16)
C41—C42—C43—C44	0.0 (3)	C7—C8—P2—C51	88.68 (13)
C42—C43—C44—C45	−0.5 (3)	C9—C8—P2—C51	−94.93 (15)
